# Propofol rather than Isoflurane Accelerates the Interstitial Fluid Drainage in the Deep Rat Brain

**DOI:** 10.7150/ijms.54320

**Published:** 2021-01-01

**Authors:** Guomei Zhao, Hongbin Han, Wei Wang, Kaiying Jia

**Affiliations:** 1 Department of Geriatrics, Beijing Shijitan Hospital, Capital Medical University, Beijing 100038, China; 2 Department of Radiology, Peking University Third Hospital, Beijing 100191, China; 3 Beijing Key Laboratory of Magnetic Resonance Imaging Technology, Beijing 100191, China; 4 Institute of Biomedical Engineering, Peking University Shenzhen Graduate School, Shenzhen 518055, China; 5 Research Institute for Translation Medicine on Molecular Function and Artificial Intelligence Imaging, Department of Radiology, The First People's Hospital of FoShan, Foshan 52800, China

**Keywords:** Propofol, isoflurane, deep rat brain, extracellular space, interstitial fluid

## Abstract

**Objective:** Different anesthetics have distinct effects on the interstitial fluid (ISF) drainage in the extracellular space (ECS) of the superficial rat brain, while their effects on ISF drainage in the ECS of the deep rat brain still remain unknown. Herein, we attempt to investigate and compare the effects of propofol and isoflurane on ECS structure and ISF drainage in the caudate-putamen (CPu) and thalamus (Tha) of the deep rat brain.

**Methods:** Adult Sprague-Dawley rats were anesthetized with propofol or isoflurane, respectively. Twenty-four anesthetized rats were randomly divided into the propofol-CPu, isoflurane-CPu, propofol-Tha, and isoflurane-Tha groups. Tracer-based magnetic resonance imaging (MRI) and fluorescent-labeled tracer assay were utilized to quantify ISF drainage in the deep brain.

**Results:** The half-life of ISF in the propofol-CPu and propofol-Tha groups was shorter than that in the isoflurane-CPu and isoflurane-Tha groups, respectively. The ECS volume fraction in the propofol-CPu and propofol-Tha groups was much higher than that in the isoflurane-CPu and isoflurane-Tha groups, respectively. However, the ECS tortuosity in the propofol-CPu and propofol-Tha groups was much smaller than that in isoflurane-CPu and isoflurane-Tha groups, respectively.

**Conclusions:** Our results demonstrate that propofol rather than isoflurane accelerates the ISF drainage in the deep rat brain, which provides novel insights into the selective control of ISF drainage and guides selection of anesthetic agents in different clinical settings, and unravels the mechanism of how general anesthetics function.

## Introduction

General anesthesia is of great significance to the clinical care of human patients 1. Nonetheless, how general anesthetics eventually induce the general anesthesia state is still unknown 2. Recent studies have found that general anesthetics can affect the brain extracellular space (ECS) and interstitial fluid (ISF) drainage, which contributes to unraveling how general anesthetics exert their functions in the brain 3,4.

The ECS is the space between adjacent neural cells in the brain, occupying approximately 15-20% of whole brain volume 5. The ECS plays a critical role in maintaining brain function and physical properties despite its irregular, tortuous, and narrow features 5-8. The ISF comprises 12-20% of whole brain water and drainages along the ECS 9. It provides a direct living microenvironment for brain cells, delivers neurotransmitters and pharmaceutical agents, and clears metabolic waste products from the brain parenchyma 5,9-11. Drugs administered into the ECS can directly bypass the blood-brain barrier and be transported along the ISF to target brain areas directly 12,13. Therefore, it is of great clinical relevance to investigate factors affecting brain ECS structure and ISF drainage.

Propofol (2, 6-diisopropylphenol) is a widely used systemic anesthetic that acts on γ-aminobutyric acid type A (GABA_A_) receptors, glycine receptors, nicotinic acetylcholine receptors, and N-methyl-D-aspartate (NMDA) receptors 14-16. Moreover, propofol can suppress locus coeruleus (LC) neuronal activity, which may contribute to the inhibition of norepinephrine (NE) release 17. Isoflurane is an inhaled anesthetic that can target various receptors like GABA_A_ receptors, and NMDA receptors to induce anesthesia 18. Isoflurane increases NE release in the deep rat brain when inhaled at a concentration of about 1.8% 4,18-20.

Anesthetic agents can differentially affect ISF drainage in the ECS of the superficial rat brain 4. However, their effects on ISF drainage in the ECS of the deep rat brain still remain largely unknown. Recently, we uncovered that while dexmedetomidine and isoflurane could induce anesthesia, dexmedetomidine markedly enhanced ISF drainage in the deep brain of rats compared to that in rats receiving isoflurane, and these phenomena can be underpinned by different NE concentrations 3. However, there is limited information on the differences of the effects of other anesthetics on ECS structure and the ISF drainage in the deep rat brain, which warrants further investigation. Considering that our previous study found that lower NE levels can enhance ISF drainage in the deep brain 3, we hypothesized that propofol could accelerate ISF drainage in the deep brain compared with isoflurane.

In the present study, tracer-based magnetic resonance imaging (MRI) and fluorescent-labeled tracer assay were employed to quantify ISF drainage in the anesthetized rodent deep brain. Pysiologic parameters of the anesthetized rats in separate groups were monitored. The caudate-putamen (CPu) and thalamus (Tha) of deep brain areas were selected for detection. Ultimately, our results provide conclusive evidence that propofol does accelerate the ISF drainage in the deep rat brain compared with isoflurane. This finding will provide novel insights to guide the selection of anesthetic agents in specific clinical settings.

## Materials and Methods

### Animal

All animal studies were approved by the Peking University Biomedical Ethics Committee (approval number: LA201461) and were performed in accordance with the Helsinki Declaration and the National Guidelines for the Care and Use of Laboratory Animals. Rats were housed individually in a 12:12 h light/dark cycle under controlled temperature (22 ± 1 °C) and humidity (60 ± 5%), with food and water available *ad libitum*.

**Figure 1:**
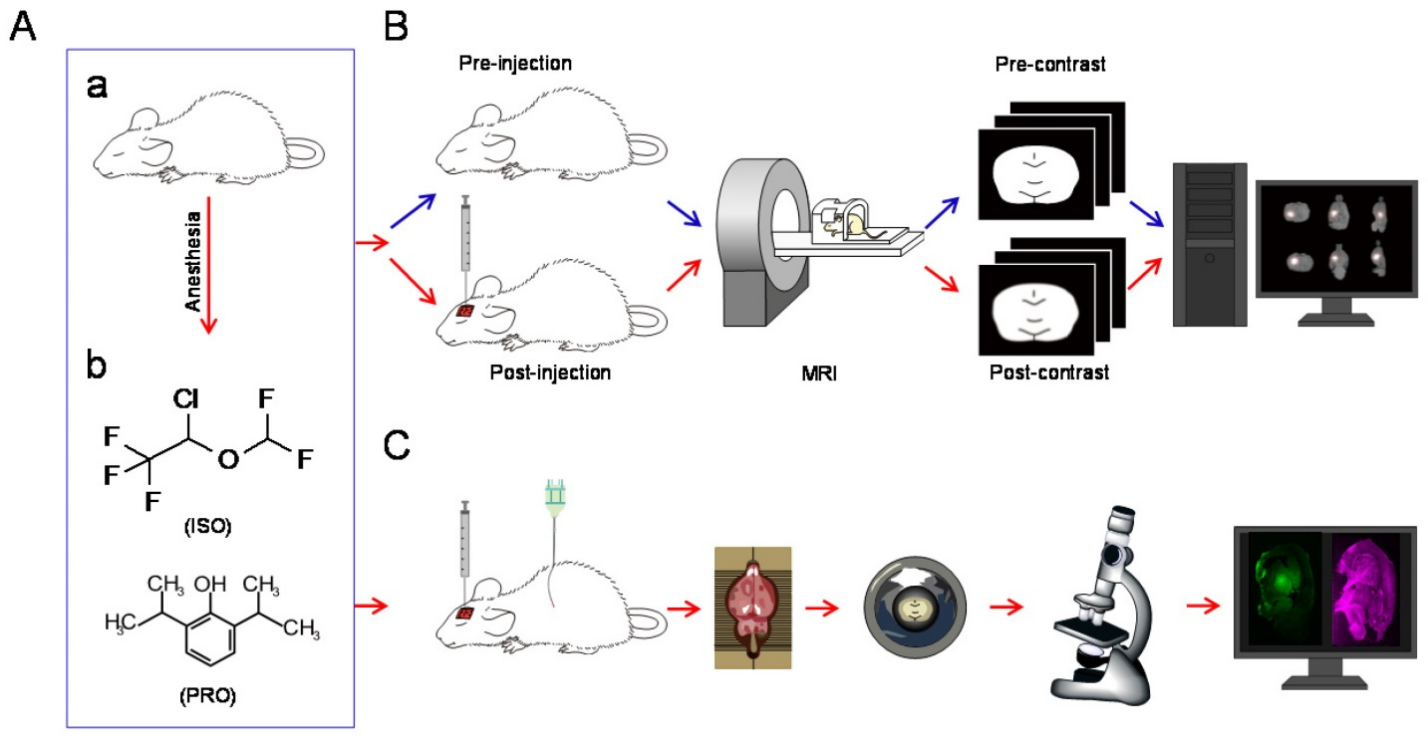
Schematic diagram of the experiments. (A) All rats were anesthetized with isoflurane (ISO) or propofol (PRO). (B) MRI was performed before and after injection of Gd-DTPA into the rat CPu or Tha. After a series of operations, post-processed MRI images were obtained 3. (C) Schematic diagram of fluorescence-based assays. After injection of LYCH into the CPu or Tha, rats were perfused and brain tissue was processed and subjected to confocal imaging.

Adult Sprague-Dawley rats (250 to 300 g) were anesthetized with isoflurane or propofol, respectively (Figure 1A). There were six rats in each group. Isoflurane (Macklin, Shanghai, China) anesthesia was started at 4% induction with 1.5 to 1.8% maintenance delivered in a 1:1 oxyen:air mixture through a tube in a breathing mask. Exhaled gas from the rats was led from another tube of the breathing mask to be actively absorbed by a gas recovery tank. In the propofol groups, rats were intraperitoneally anesthetized with 60 mg/kg propofol (AstraZeneca S.p.A., Milan, Italy) and maintained with a continuous infusion of 0.6 mg/kg/min delivered via a subcutaneous catheter. Rats in the propofol anesthesia groups were exposed to the same 1:1 oxyen:air mixture as the isoflurane group. The subsequent experiments were conducted when rats lost the righting reflex. The two anesthetics were used in an equipotent dose/concentration during the experiments. A heating pad was used to maintain the body temperature of rats at 37 ± 0.2 °C.

### Tracer Injections into the Rat Brain

Gadolinium-diethylenetriamine penta-acetic acid (Gd-DTPA, 10 mM, Magnevist; Bayer Schering Pharma AG, Berlin, Germany), which cannot be taken up by neural cells, was prepared as described previously 6. The dipotassium salt of LYCH (lucifer yellow CH) (10 mM, Glentham Life Sciences Ltd, Wiltshire, United Kingdom) was used as the fluorescent-labeled tracer in this study.

Anesthetized rats were fixed in a stereotaxic apparatus (Lab Standard Stereotaxic-Single; Stoelting Co., Wood Dale, IL, USA) with either of the following: 1) Gd-DTPA (2 µL, 10 mM); 2) LYCH (2 µL, 10 mM) injected into the CPu (AP: 0mm, L: -2.8 mm, V: -5.8 mm) or Tha (AP: -3 mm, L: -3 mm, V: -6 mm) 21. After injection, the needle was left in place for 10 min before removal to prevent the tracer from retracting along the needle path.

### Tracer-based MRI Scan Protocols and Post-Procedure Image Processing

Image analysis and post-procedure image processing were performed as described previously 10,22,23. For each rat, MRI scanning was performed before and after Gd-DTPA injection (Figure 1B). Time points of scanning were set at pre-injection and post-injection until the “bright region” faded. All post-injection images were subjected to rigid transformation, similarity measurements, high-order interpolation, and adaptive stochastic gradient descent optimization (Figure 1B). These images were then subtracted from the pre-scanned images. The acquired “bright areas”, which were obtained by establishing a seed point and a threshold in the regions of interest, were assumed to be related to the presence of the tracer. New sets of post-processed MRI images in the horizontal, sagittal, and coronal planes with slice thicknesses of 1 mm were generated by the software. The signal intensity within the target area of the processed images was measured and denoted by ΔSI after the co-registration and subtraction process, which can be used to calculate diffusion parameters in the rat brain ECS.

### Calculation of ECS Structural Parameters and ISF Diffusion Parameters

The ECS structural parameters and ISF diffusion parameters were analyzed with the NanoDetect Analyze system (MRI lab; Beijing, China, version 2.1) as described previously 10,24.

### Measurement of Physiologic Parameters

Heart rate, systolic pressure, respiratory rate, and noninvasive oxygen saturation (SaO_2_) in rats were measured by noninvasive monitors (Smiths Medical ADS. Inc.). In these experiments, Gd-DTPA was also infused into the rats' right CPu or Tha (n=6 per group). Heart rate, systolic pressure, respiratory rate, and SaO_2_ were acquired every 20 min for 2 h. The caudal artery and hind paws of rats were measured to acquire systolic pressure and SaO_2_, respectively.

### Fluorescent-labeled Tracer Assay

1.5 h after stereotaxic injection of LYCH, the rats were sacrificed and intracardially perfused with 0.9% saline followed by 4% paraformaldehyde (PFA) for 15-20 min. The brains were then excised and post-fixed in 4% PFA overnight. Next, sagittal position brain slices with a thickness of about 1mm, which can facilitate simultaneous viewing of the CPu and Tha in the same slice, were obtained using a rat brain mold. Then, the slices were put onto glass slides, mounted with coverslips, and examined immediately with a confocal laser scanning microscope (TCS SP8 MP, Leica, Germany) using excitation at 488 nm and reflection at 633 nm under the 25 × objective with additional zooming. Image acquisition and data processing were performed under the same conditions and the maximum distribution area of LYCH in each slice was calculated (Figure 1C).

### Statistical Analysis

Statistical analyses of the present study were performed using IBM SPSS software, version 23.0 (IBM Corp, Armonk, NY). Data are expressed as the mean ± standard error. One-way analysis of variance with Duncan's *post-hoc* tests (overall significance level = 0.05) was used to compare ECS structural parameters, ISF drainage parameters, the maximum distribution area of LYCH, and physiologic parameters. P-value < 0.05 was considered statistically significant.

## Results

### ISF Drainage in Propofol -Anesthetized and Isoflurane -Anesthetized Rats

**Figure 2:**
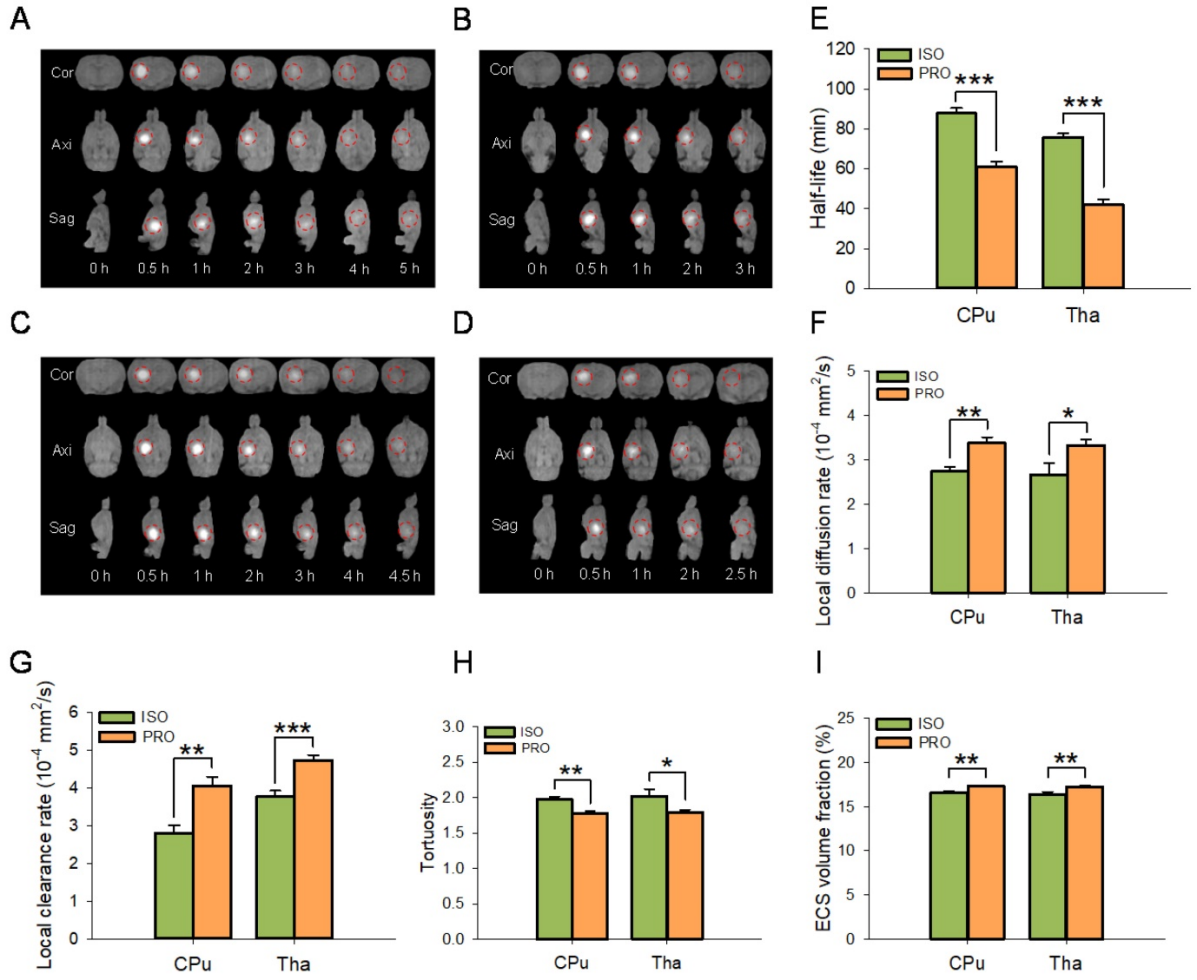
PRO enhanced ISF drainage in the ECS of the CPu and Tha in rats compared with ISO. (A-D) Drainage of ISF visualized using the tracer-based MRI showing Cor, Axi, and Sag planes. (A-B) Drainage of ISF in CPu of rats anesthetized with ISO or PRO, respectively. (C-D) Drainage of ISF in Tha of rats anesthetized with ISO or PRO, respectively. Compared with ISO, PRO significantly decreased the half-life of ISF (E) and increased the diffusion rate (F) and clearance rate of ISF (G) in the CPu and Tha. Compared to ISO, PRO significantly decreased tortuosity (H) but increased the volume fraction of the ECS (I). Data are expressed as the mean ± standard error. Scale bars, 1.5 cm in A-D. Asterisks indicate significant differences between samples (*, P < 0.05; **, P < 0.01; ***, P < 0.001; Duncan's test; n = 6). Abbreviations: Axi: axial; Cor: coronal; Sag: sagittal.

ECS structure and ISF drainage were significantly different among rats anesthetized with propofol or isoflurane (Figure 2A-I). MRI results showed that the half-life of the ISF was significantly decreased in propofol-anesthetized rats compared to that in isoflurane-anesthetized rats in both the CPu (60.80 ± 2.61 min vs 87.78 ± 2.69 min, P < 0.001; Duncan's tests; n = 6) and Tha (41.86 ± 2.79 min vs 75.86 ± 1.83 min, P < 0.001; Duncan's tests; n = 6) groups (Figure 2E). The local diffusion rate was significantly higher in propofol-anesthetized rats than in isoflurane-anesthetized rats in both the CPu (3.38 ± 0.13×10^-4^ mm^2^/s vs 2.74 ± 0.11×10^-4^ mm^2^/s, P < 0.01; Duncan's tests; n = 6) and the Tha (3.33 ± 0.14×10^-4^ mm^2^/s vs 2.67 ± 0.26 ×10^-4^ mm^2^/s, P < 0.05; Duncan's tests; n = 6) groups (Figure 2F). Similarly, the local clearance rate of ISF was significantly higher in propofol-anesthetized rats than in isoflurane-anesthetized rats in both the CPu (4.05 ± 0.23×10^-4^ mm^2^/s vs 2.80 ± 0.21×10^-4^ mm^2^/s, P < 0.01; Duncan's tests; n = 6) and the Tha (4.73 ± 0.13×10^-4^ mm^2^/s vs 3.77 ± 0.16 ×10^-4^ mm^2^/s, P < 0.001; Duncan's tests; n = 6) groups (Figure 2G).

For ECS structural parameters, tortuosity in the brains of propofol-anesthetized rats was dramatically decreased compared to that in isoflurane-anesthetized rats in both the CPu (1.77 ± 0.03 vs 1.97 ± 0.04, P < 0.01; Duncan's tests; n = 6) and the Tha (1.79 ± 0.04 vs 2.02 ± 0.10, P < 0.05; Duncan's tests; n = 6) groups (Figure 2H). ECS volume fraction was significantly higher in the propofol-anesthetized rats than in the isoflurane-anesthetized rats, both in the CPu (17.28 ± 0.13% vs 16.61 ± 0.11%, P < 0.01; Duncan's tests; n = 6) and the Tha (17.23 ± 0.10% vs 16.39 ± 0.27%, P < 0.01; Duncan's tests; n = 6) groups (Figure 2I).

### ISF Drainage in the CPu and Tha

**Figure 3:**
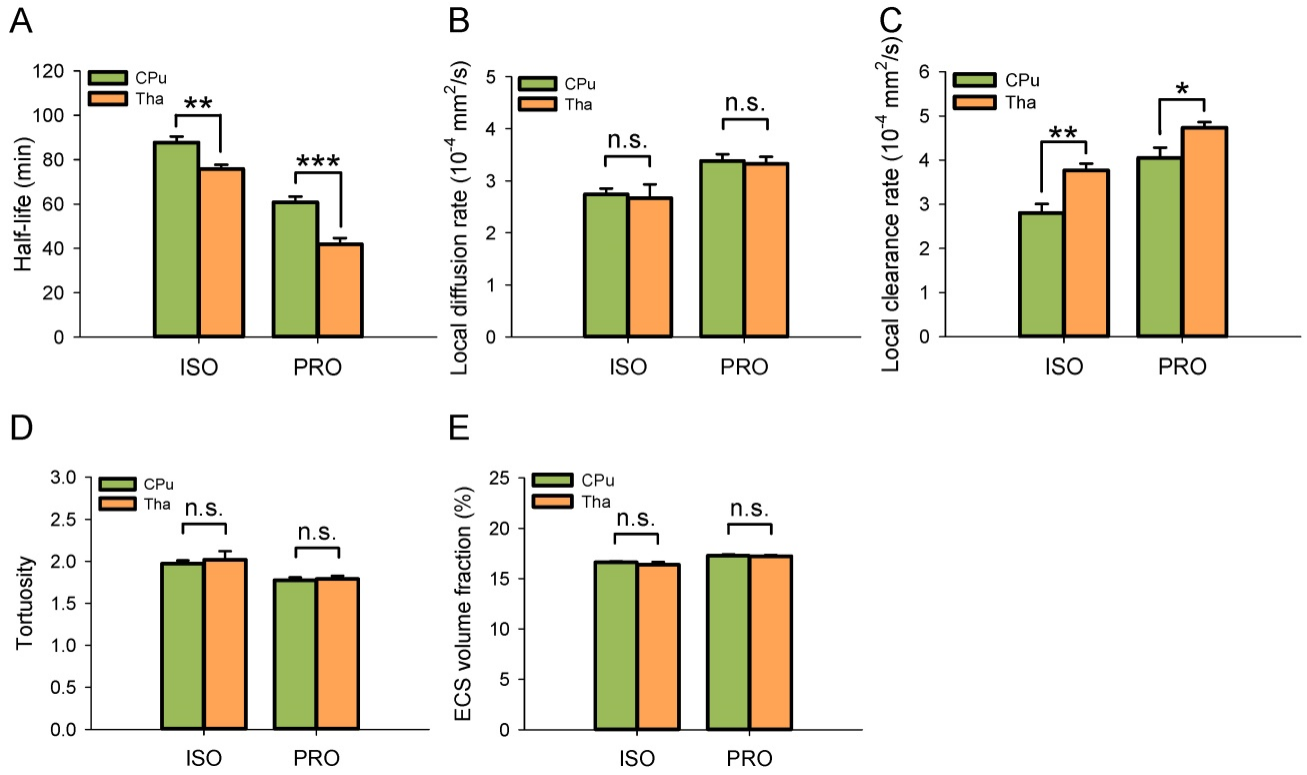
ISF drained faster in the Tha than in the CPu under the same anesthetic agent. (A) The half-life of ISF in the Tha decreased markedly either under ISO or PRO anesthesia compared to that in the CPu. (B) There was no significant difference in the local diffusion rate of ISF between the Tha and CPu under ISO or PRO. (C) The local clearance rate of ISF was higher in the Tha than in the CPu under ISO or PRO. (D) There was no significant difference in tortuosity between the Tha and CPu under ISO or PRO. (E) There was no significant difference in the ECS volume fraction between the Tha and CPu under ISO or PRO. Asterisks indicate significant differences between samples (*, P < 0.05; **, P < 0.01; ***, P < 0.001; Duncan's test; n = 6).

Despite using the same anesthetic agent, the half-life of ISF in the Tha decreased significantly under both isoflurane (75.86 ± 1.83 min vs 87.78 ± 2.69 min, P < 0.01; Duncan's tests; n = 6) and propofol anesthesia (41.86 ± 2.79 min vs 60.80 ± 2.61 min, P < 0.001; Duncan's tests; n = 6) compared with the CPu (Figure 3A). The local clearance rate was significantly higher in the Tha than in the CPu under isoflurane (3.77 ± 0.16×10^-4^ mm^2^/s vs 2.80 ± 0.21×10^-4^ mm^2^/s, P < 0.01; Duncan's tests; n = 6) and propofol anesthesia (4.73 ± 0.13×10^-4^ mm^2^/s vs 4.05 ± 0.23×10^-4^ mm^2^/s, P < 0.05; Duncan's tests; n = 6) (Figure 3B). There were no significant differences in the local diffusion rate, tortuosity, or ECS volume fraction between the CPu and Tha under the same anesthetic agent. These results were in congruence with our previous study 10.

### Effects of Propofol and Isoflurane on the Vasomotor Function of Rats

ISF drainage is closely related to the cardiac cycle and respiratory cycle-associated pulsatility 25. In this regard, anesthetics could have a significant effect on vasomotor function. Thus, we monitored the heart rate, respiratory rate, systolic pressure, and SaO_2_ (%) in rats anesthetized with propofol and isoflurane 4,26. The heart rate of rats with operation in the CPu under the two different anesthetics displayed no significant differences (P > 0.05; Duncan's tests; n = 6) (Table 1). Respiratory rate, systolic pressure, and SaO_2_ (%) also showed no significant differences (P > 0.05; Duncan's tests; n = 6) (Table 1), respectively. Furthermore, we observed similar heart rate, respiratory rate, systolic pressure, and SaO_2_ in rats with operation in the Tha anesthetized with the two different anesthetics and in rats with operations in CPu or Tha under the same anesthetic agents. Accordingly, the effect of the anesthetics on heart rate, respiratory rate, systolic pressure, and SaO_2_ (%) were unlikely to influence the results of the tracer-based MRI studies.

### Propofol Can Accelerate the ISF Drainage

**Figure 4:**
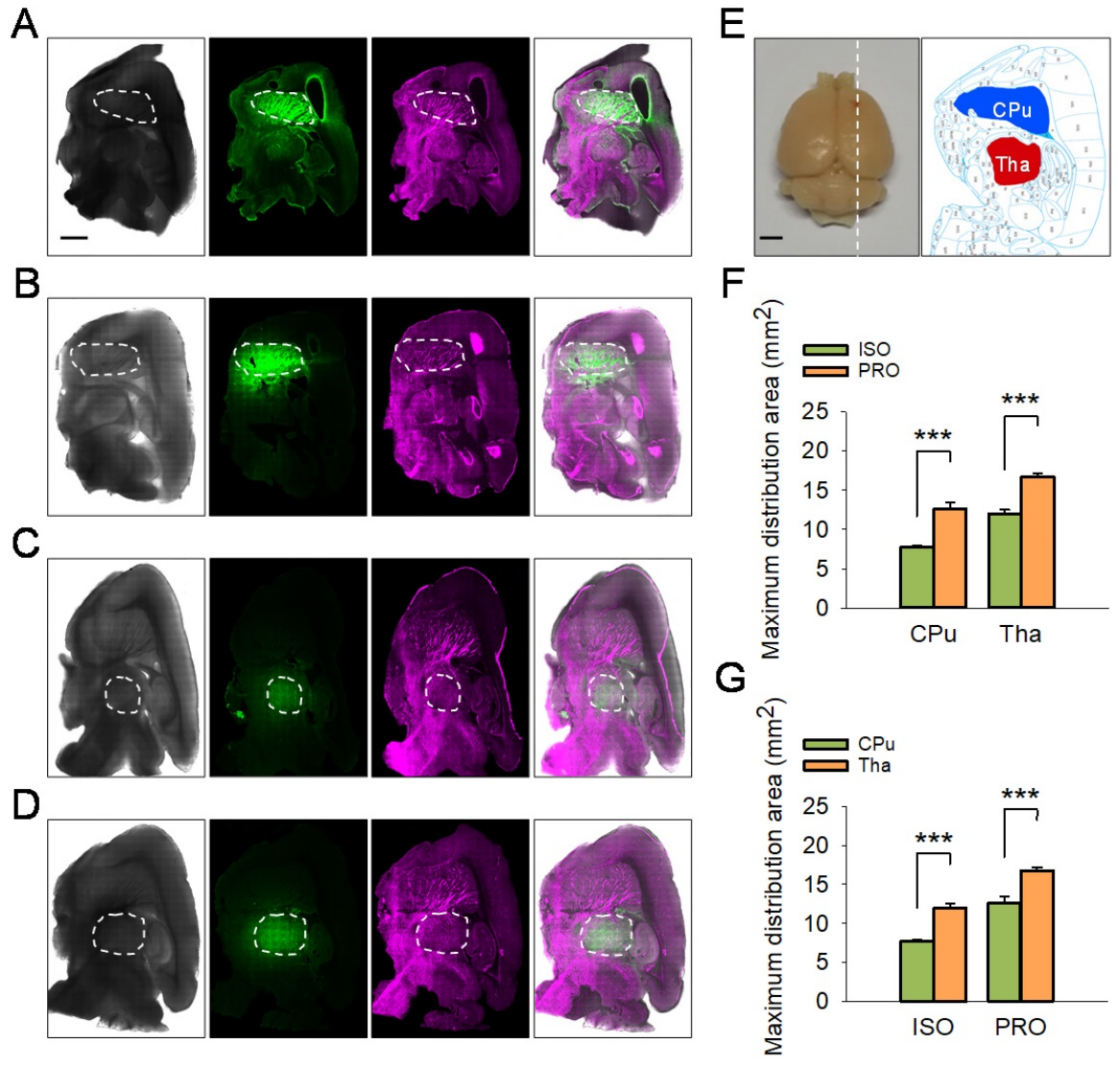
The maximum distribution area of LYCH in ISO-anesthetized rats was smaller compared to PRO-anesthetized rats. (A-D) Reflected light images with excitation at 488 nm and images reflected at 633 nm. Overlaid fluorescent images and reflected light images are shown on the right (25 × magnification). The white dashed box indicates the maximum distribution area of LYCH. (A-B) The maximum distribution area of LYCH in CPu slices in ISO and PRO groups. (C-D) The maximum distribution area of LYCH in Tha slices in ISO and PRO groups. (E) The diagram on the left shows a rat brain; white dotted line indicates the direction of sagittal brain slices. The diagram on the right presents sagittal brain slices, including the CPu (blue area) and Tha (red area). (F) The maximum distribution area of LYCH in ISO groups was smaller than in PRO groups. (G) Under the same anesthetic, the maximum distribution area of LYCH was larger in Tha slices than in CPu slices. Scale bar: 2 mm. Data are expressed as mean ± standard error. Asterisks indicate significant differences between samples (*, P < 0.05; **, P < 0.01; ***, P < 0.001; Duncan's test; n = 6).

The tracer-based MRI studies described above suggest propofol can accelerate ISF drainage in the deep rat brain compared with isoflurane. To corroborate this phenomenon, we conducted fluorescent-labeled tracer studies. After the rats were anesthetized with propofol or isoflurane, LYCH was injected into the CPu or the Tha of rats as noted above (Figure 4A-E) and the maximum distribution area of LYCH in each sagittal position brain slice was calculated and compared among the groups (Figure 4). In line with the tracer-based MRI studies, the maximum distribution area of LYCH in the propofol groups was much larger than that in the isoflurane groups both for CPu (12.58 ± 0.82 vs 7.71 ± 0.27, P < 0.001; Duncan's tests; n = 6) and Tha (16.75 ± 0.46 vs 11.97 ± 0.59, P < 0.001; Duncan's tests; n = 6) slices (Figure 4F). These results are consistent with the fluorescence and reflected light charts as well as the overlay charts of the two, where the maximum distribution area of LYCH in propofol is much larger than that of the isoflurane groups in both the CPu and Tha slices (Figure 4A-D). Similar to the tracer-based MRI results, the maximum distribution area of LYCH was much smaller in CPu sagittal brain slices compared to Tha under both isoflurane (7.71 ± 0.27 vs 11.97 ± 0.59, P < 0.001; Duncan's tests; n = 6) and propofol anesthesia (12.58 ± 0.82 vs 16.75 ± 0.46, P < 0.001; Duncan's tests; n = 6) (Figure 4G).

## Discussion

In this study, we observed that propofol significantly decreased tortuosity, increased ECS volume fraction, and accelerated ISF drainage in the deep rat brain when compared to isoflurane. ISF drainage is closely related to the cardiac cycle and respiratory cycle-associated pulsatility 25, and we record similar physiological parameters in rats anesthetized with propofol and isoflurane. Therefore, propofol and isoflurane cannot induce the tracer-based MRI and fluorescent-labeled tracer studies through influencing the vasomotor function in rats.

Propofol is a widely used intravenous anesthetic which can interact with GABA_A_ receptors, glycine receptors and other neurotransmitter receptors to induce sedation and unconsciousness 15,17. Moreover, propofol can decrease NE release by suppressing LC neuronal activity 17,27,28. Isoflurane can bind to various receptors in the brain, including GABA_A_ and NMDA receptors, to inhibit their function and induce alterations in behavioral and physiological states 2. In contrast to propofol, isoflurane may induce NE release 18,19,29-32. Indeed, the LC-NE system may indicate the specific mechanism of how NE levels change under different types of anesthesia. A previous study showed that propofol could suppress the spontaneous firing of LC neurons by increasing GABAergic inputs to these cells whereas the inhalational anesthetics may activate LC neurons by a gap junction-related mechanism 17.

**Figure 5:**
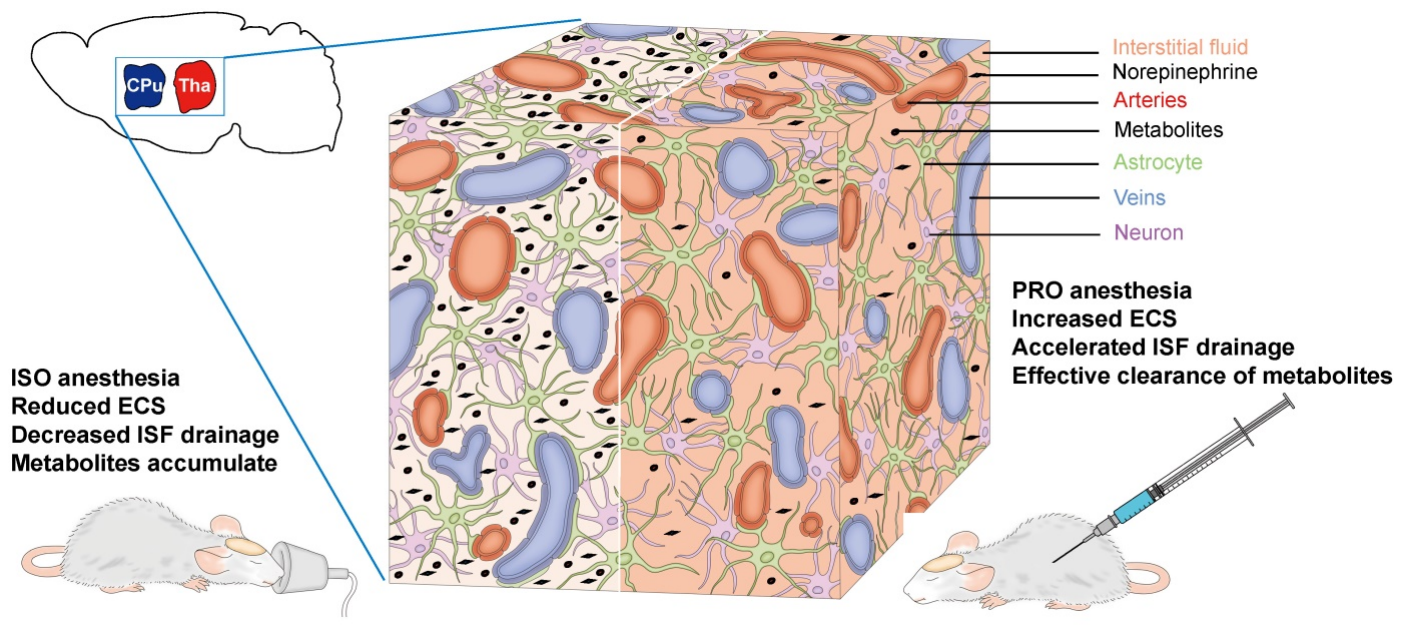
Schematic diagram of the effects of NE on the ECS and ISF in the deep rat brain. ISO increases the NE release in the CPu and Tha, leading to a decreased ECS volume fraction, decreased ISF drainage and eventual accumulation of metabolites. PRO can decrease NE release and increase ECS volume fraction, resulting in accelerated ISF drainage and more effective clearance of metabolites.

The differences in drainage of the ISF we observed could be attributable to the various effects of the two anesthetic agents on the release of NE, since NE levels are closely related to ISF drainage 3,33. Therefore, propofol anesthesia, which could induce a lower NE level in the rat brain, would induce accelerated ISF drainage and more effective clearance of metabolites when compared with isoflurane anesthesia (Figure 5). Furthermore, isoflurane, as a volatile general anesthetic, may exert neurotoxic effects, which can alter ECS structure and slow ISF drainage 34. Our findings could be helpful in selecting the appropriate anesthetic when clinical control over clearance of metabolic waste products and the transportation of pharmaceutical agents in the brain is required. Although the precise molecular mechanisms underlying the role of NE in ECS structure and ISF drainage still remain to be elucidated and the anesthetic dosages in this study cannot be easily extrapolated to clinical use, our study is invaluable and meaningful for anesthetics choices in clinical settings.

Collectively, in the present study, we demonstrated that propofol can significantly accelerate the ISF drainage in deep rat brain compared to isoflurane. The clinical implications of our study require further investigation.

## Figures and Tables

**Table 1:** Physiologic parameters in rats anesthetized with PRO and ISO with operations performed in the CPu and Tha.

Physiologic parameters	Anesthesia	Brain Areas	20 min	40 min	60 min	80 min	100 min	120 min
Heart Rate (bpm)	Isoflurane	CPu (n = 6)	343.33 ± 2.75	342.00 ± 1.70	341.50 ± 1.21	343.17 ± 2.25	343.00 ± 3.27	345.33 ± 2.04
		Tha (n = 6)	345.17 ± 2.31	345.17 ± 2.01	344.00 ± 2.02	343.17 ± 2.35	343.50 ± 2.56	342.00 ± 2.45
	Propofol	CPu (n = 6)	344.33 ± 2.23	341.33 ± 1.47	345.67 ± 2.42	344.33 ± 2.27	342.67 ± 3.14	344.67 ± 2.93
		Tha (n = 6)	344.00 ± 3.03	343.50 ± 2.42	344.50 ± 2.14	342.67 ± 2.80	345.50 ± 3.86	342.67 ± 3.57
Respiratory Rate (bpm)	Isoflurane	CPu (n = 6)	53.33 ± 0.93	54.50 ± 1.97	54.50 ± 1.64	52.50 ± 1.21	53.17 ± 0.97	52.67 ± 1.08
		Tha (n = 6)	53.00 ± 1.10	54.00 ± 1.79	53.33 ± 0.98	54.00 ± 0.95	53.67 ± 1.78	54.33 ± 1.57
	Propofol	CPu (n = 6)	52.17 ± 0.86	52.83 ± 1.16	54.83 ± 1.72	52.00 ± 0.89	52.50 ± 1.33	52.83 ± 1.16
		Tha (n = 6)	52.33 ± 0.93	53.17 ± 1.39	54.67 ± 1.33	54.33 ± 1.40	55.50 ± 1.37	53.33 ± 1.08
Systolic Pressure (mmHg)	Isoflurane	CPu (n = 6)	124.33 ± 2.31	124.53 ± 2.81	125.43 ± 3.49	125.13 ± 4.87	126.05 ± 3.38	124.37 ± 4.42
		Tha (n = 6)	127.45 ± 1.19	123.35 ± 2.66	124.27 ± 1.99	125.90 ± 1.67	126.41 ± 5.30	124.70 ± 2.65
	Propofol	CPu (n = 6)	125.84 ± 2.30	127.26 ± 3.44	121.56 ± 5.20	120.45 ± 2.16	120.63 ± 5.92	122.04 ± 3.69
		Tha (n = 6)	126.81 ± 2.12	125.71 ± 2.63	124.48 ± 1.94	126.87 ± 1.21	125.38 ± 0.98	125.49 ± 2.09
SaO_2_ (%)	Isoflurane	CPu (n = 6)	94.17 ± 1.46	93.33 ± 2.25	90.17 ± 1.72	90.50 ± 1.21	91.00 ± 1.67	91.33 ± 1.17
		Tha (n = 6)	93.83 ± 1.53	90.67 ± 2.25	91.83 ± 1.66	90.83 ± 1.32	92.83 ± 1.53	92.17 ± 1.46
	Propofol	CPu (n = 6)	93.83 ± 2.29	93.33 ± 1.75	94.33 ± 1.40	91.67 ± 2.27	91.67 ± 1.40	91.50 ± 1.67
		Tha (n = 6)	92.67 ± 1.72	91.17 ± 1.39	90.67 ± 1.17	92.17 ± 2.04	93.17 ± 0.74	93.17 ± 1.59

The heart rate, respiratory rate, systolic pressure, and SaO_2_ (%) in rats in the CPu or Tha injection groups under the two different anesthetics display no significant differences (P > 0.05; Duncan's test).

## Abbreviations

CPu: caudate-putamen
ECS: extracellular space
GABA_A_: γ-aminobutyric acid type A
ISF: interstitial fluid
LC: locus coeruleus
LYCH: Lucifer Yellow CH
MRI: Magnetic resonance imaging
NE: norepinephrine
NMDA: N-methyl-D-aspartate
PFA: paraformaldehyde
PRO: propofol
ISO: isoflurane
SaO_2_: noninvasive oxygen saturation
Tha: thalamus
